# The effect of subjective life expectancy on the participation in commercial pension insurance of Chinese elderly

**DOI:** 10.3389/fpsyg.2022.969719

**Published:** 2022-08-22

**Authors:** Mei Zhou, Yingyi Wang, Yunjia Liang, Ruonan Shi, Shaoyang Zhao

**Affiliations:** ^1^School of Public Administration, Southwestern University of Finance and Economics, Chengdu, China; ^2^School of Economics, Sichuan University, Chengdu, China

**Keywords:** subjective life expectancy, commercial pension insurance, elderly, heterogeneity, IV method

## Abstract

**Objective:**

This paper studies the impact of the subjective life expectancy of the elderly on their commercial pension insurance participation at micro perspective, providing new evidence to explain the motivation of commercial pension insurance participation in China.

**Methods:**

Using 4 waves of data from the China Health and Retirement Longitudinal Study (CHARLS) from 2011 to 2018, a multiple linear regression model is constructed to investigate the effect of subjective life expectancy on commercial pension insurance participation among the Chinese elderly, and IV model estimation shows that the results are robust. Meanwhile, the heterogeneity of the effect of elderly life expectancy on commercial pension insurance participation behavior among different characteristic groups is also studied.

**Results:**

A one standard deviation improvement in our measure of subjective life expectancy predicts a 0.9% point higher participation rate in commercial pension insurance. We also find that there is significant heterogeneity in the effects of subjective life expectancy on the participation of elderly people in commercial pension insurance with respect to gender, education, hukou, and wealth.

**Conclusion:**

This paper provides a new perspective to explain the factors influencing commercial pension insurance participation in China. We suggest that improving residents’ awareness of life expectancy is beneficial to their reasonable retirement planning, in the background of stepping into an aging society in China.

## Introduction

**Figure 1:**
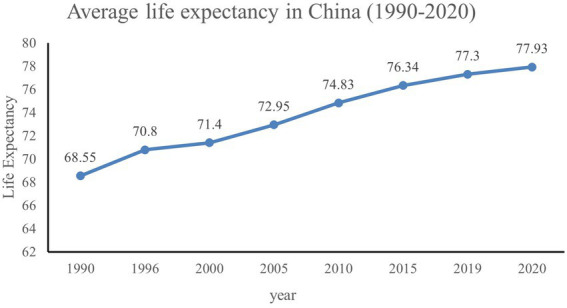
Average life expectancy in China (1990–2020). Data obtained through the National Bureau of Statistics.

The health status of the Chinese population has continued to improve over the decades, with the average life expectancy increasing from 35 years in the 1950s to 77.9 years in 2020 (Figure 1). It is expected that in 2035, the average life expectancy in China will be 80 years or more. At the same time, the aging of the Chinese population is becoming increasingly serious. The population of people aged 65 and above increased from 63.0 million to 200.51 million from 1990 to 2021, accounting for 14.2% of the total population, up from 5.57%, which means that China is stepping into an aging society. Based on China’s demographic structure, it is speculated that the aging population of China will further increase in the coming decades. How to deal with the personal financial risks associated with increased lifespan will be an important issue for older people in China. Currently, the development of China’s pension system is mainly focused on the first and second pillars. Among them, basic pension insurance, accounting for 70%, enterprise annuities account for 29%, while the individual commercial pension insurance is seriously lagging. Data show that the replacement rate of basic pension insurance is only about 40% at present, and the issue of pension security should be taken seriously. Therefore, the Chinese government encourages residents to actively participate in commercial pension insurance to increase the replacement rate of residents after retirement and improve the life quality of the elderly. This paper focuses on the effect of subjective life expectancy on commercial insurance participation.

Social security in China is dominated by public insurance, and the development of private insurance lags behind. The participation rate of residents in commercial insurance is low because of their poor insurance consciousness. Past research provides considerable evidence of factors that influence the willingness to purchase commercial insurance, mainly in terms of macro-environmental and micro-personal dimensions. A substantial literature finds that income significantly affects residents’ commercial insurance purchasing behavior. The demand for commercial life insurance among residents increases significantly as income and household wealth increase (Hammond et al., 1967; Browne and Kim, 1993; Thorsten and Ian, 2003) Macro factors such as *per capita* disposable income, saving rate, and coverage of social insurance are also important factors influencing residents’ commercial insurance purchases. In recent years, more research uses micro data to investigate the influence of individual characteristics on the demand for commercial insurance, which include age, gender, marital status, and family size (Kalar and Shukla, 2010). In the traditional Chinese culture, there is another concept, bringing up children to support parents in their old age that has an impact on residents’ pension reserve. This concept is more widespread in rural areas. Therefore, the number of children and the region where they live can also have an impact on residents’ commercial pension insurance decisions (Zhang and Tang, 2021). However, there is less literature studying the impact of life expectancy on commercial pension insurance participation.

Subjective life expectancy is an individual’s subjective perception or subjective prediction of the length of life and is mainly influenced by characteristics such as gender, lifestyle habits, and length of parental survival (Hanna and Kène, 2010; O’Connell, 2011; Myrseth et al., 2019; Philipov and Scherbov, 2020). Subjective life expectancy affects individuals’ behavior decisions, such as insurance purchases, retirement age, consumption levels, and savings rates. Overoptimistic or overpessimistic subjective expectations can lead to suboptimal behavioral decisions (Cutler et al., 2008). Zhou et al. (2015) finds no significant effect of subjective survival probability on whether rural residents participate in China’s new rural pension insurance for individuals, but residents with higher subjective survival probability are more likely to choose higher premium levels. The earliest study that shows the effect of life expectancy on residents’ property allocation is the life-cycle theory that has been used until now, which is the theoretical basis for many economists to explain the correlation between age structure and residents’ savings and consumption. For example, Lee et al. (1998) find that longer life expectancy leads residents in East Asia to anticipate longer periods of consumption after retirement and causes a significant increase in the savings rate in the region. However, as the economy in the United States grows, the health of the population improves, life expectancy increases, and residents work for a correspondingly longer period of time, which leads to a lower saving rate and thus higher consumption instead (Fogel, 1991). Rational consumers tend to choose to work longer in response to longer life expectancy so they can earn more without reducing their current consumption and have more savings to sustain their retirement life in old age, thus longer life expectancy mainly affects the retirement decision, while there may not be a significant effect on residents’ consumption (Kalemli-Ozcan and Weil, 2002). Using data from the World Health Organization, Li finds that “unhealthy” life expectancy reduces the national saving rate, while “healthy” life expectancy does not have a significant effect on the national saving rate (Li, 2020).

The analysis of subjective life expectancy on asset allocation in the existing literature focuses on saving and consumption behavior. The literature explains the U-shaped puzzle of Chinese household saving rate from various perspectives such as income uncertainty (Chamon et al., 2013; Ma and Zhou, 2014), inadequate social security (Yang and Chen, 2009; Chamon and Prasad, 2010), family structure (Li and Wu, 2014), motivation of bequest (Huang and Sun, 2005; Cai and Zhang, 2020), uncertainty of lifespan (Thomas and Katja, 2013), and preventive saving (Dobrescu, 2015). In contrast, insurance has not been adequately studied as an important tool for asset allocation. This paper instead focuses on how individual life expectancy affects commercial pension insurance participation and provides new micro evidence on the commercial pension insurance participation behavior of Chinese residents.

## Materials and methods

### Data source and variable descriptions

Four waves of data from the China health and retirement longitudinal study (CHARLS) for 2011, 2013, 2015, and 2018 are used in this paper. The survey is part of a series of international health and retirement surveys conducted in the United States (HRS), United Kingdom (ELSA), South Korea (KLOSA), Europe (SHARE), and other countries and regions. CHARLS surveyed information on personal characteristics, family structure, health status, healthcare, insurance, work, retirement, pension, income, and assets of people aged 45 and above in China. Samples were drawn through four stages: county sampling, village/household sampling, household sampling, and individual sampling. The national baseline survey was conducted in 2011–12, with wave 2 in 2013, wave 3 in 2015, and wave 4 in 2018. The baseline survey covered 28 provinces, 150 counties, and 17,708 individuals in 10,257 households across the country. The follow-up surveys included return visits to the baseline survey sample and interviews with new entrants.

There is a small portion of the sample under the age of 45 because CHARLS not only interviews residents of appropriate age (45 and older), but also collects personal information from their spouses, whose age is not restricted. Eligibility for commercial pension insurance has age restrictions in China, generally requiring beneficiaries younger than 60, and only a very small number of products can be extended to age 65. Therefore, the survey sample above 60 is excluded from our study. We removed samples with missing data on subjective life expectancy, and we also excluded samples with serious missing data on basic personal information.

### Variables

The dependent variable is whether the individual has commercial pension insurance, which is a dummy variable. The key independent variable is subjective life expectancy. In this paper, subjective probability of survival is used to measure an individual’s subjective life expectancy. CHARLS asked respondents about the likelihood of envisioning themselves living to a certain target age.^1^ The target survival age for respondents aged 60 years and younger is 75 years. Respondents were asked to choose from a total of 5 options from 1 (almost impossible) to 5 (simply certain), We assigned answers 1 to 5 to correspond to 0, 25, 50, 75, and 100% likelihoods, respectively.

We control for variables that affect both the respondent’s personal subjective life expectancy and their participation in commercial insurance as much as possible, including the respondent’s age, gender, hukou, education, and marital status (Yang and Song, 2022), whether they have public pension insurance, number of surviving children (Zhang and Tang, 2021; Liu, 2022), and total household assets (Table 1). Among these variables, the dummy variable of whether having public pension insurance controls for the effect of the social security system on commercial pension insurance participation (Yang and Chen, 2009; Yin and Yan, 2020). Social pension plans, such as the New Rural Social Pension Insurance program, have a positive income effect, improving the health of participants while having an impact on participants’ retirement decisions (Shu, 2018; Cheng et al., 2018a,b; Chen et al., 2020; Caro and Parada-Contzen, 2022).

**Table 1:** Definition of variables.

Variables		Definition	Mean
Dependent variable	Participation of commercial pension insurance	Whether the respondent has commercial pension insurance (yes = 1; no = 0)	0.054
Key variable	Subjective life expectancy	Individual subjective life expectancy (equal to the subjective probability of survival answered by the respondent in the questionnaire)	0.550
Control variables	Age	Age of respondents	52.243
	Male	Gender of respondents (male = 1; female = 0)	0.460
	Hukou	Hukou type of respondents (urban = 1; rural = 0)	0.202
	Education	Education level of respondents (primary school or below = 1; junior high school = 2; senior high school = 3; college or above = 4)	1.556
	Spouse	Whether the respondent has a spouse living with him/her (yes = 1; no = 0)	0.942
	Public pension insurance	Whether the respondents have public pension insurance	0.852
	Children	Number of surviving children	2.146
	Wealth	Total household assets of respondents (logarithm)	10.555

### Model settings

OLS model is used to test the impact of subjective life expectancy of the elderly on their participation in commercial pension insurance. We first estimate the following equation.


$$Y_{ijt}=\beta_{0}+\beta_{1}L_{ijt}+\beta_{2}X_{ijt}+\gamma_{t}+\mu_{j}+\varepsilon_{ijt}$$

Where, the dependent variable represents the participation of commercial pension insurance of individual *i* in province *j* at year *t*. The $L_{ijt}$ represents the subjective life expectancy of individual *i* in province *j* at year *t*, which is measured by the subjective survival probability answered by the respondents in the questionnaire. Represents a set of control variables at the individual and household level, including the age, gender, hukou, education, spouse, whether to have public pension insurance, number of surviving children, number of family members, and household assets. We also control for year fixed effects and the region fixed effects, among them, the parameter of $L_{ijt}$ is the estimator we care about most.

## Results

### Empirical results analysis

The results of multiple linear regressions predicting the effect of subjective life expectancy of the elderly on their commercial pension insurance participation. Table 2 reports the regression results based on the OLS model estimation. Columns (1) to (4) progressively include control variables. The results show that subjective life expectancy has a significant effect on commercial pension insurance participation, and the regression results are robust. The regression results in column (4) show that one standard deviation increase in subjective life expectancy leads to a significant increase in commercial pension insurance participation by 0.9% point. This shows that the subjective life expectancy of the elderly significantly affects commercial pension insurance participation, and those who expect to live longer are more incentivized to allocate assets and save preventively for old age in advance through commercial pension insurance.

**Table 2:** Effect of subjective life expectancy on commercial pension insurance participation.

	Commercial pension insurance participation			
	(1)	(2)	(3)	(4)
Subjective Life Expectancy	0.017^***^ (0.000)	0.012^***^ (0.003)	0.009^***^ (0.003)	0.009^***^ (0.003)
Age		−0.000 (0.000)	−0.000 (0.000)	−0.000 (0.000)
Male		−0.003 (0.002)	−0.004^*^ (0.002)	−0.003 (0.002)
Hukou		0.008^***^ (0.003)	0.005 (0.003)	0.004 (0.003)
Public pension insurance		0.001 (0.002)	0.001 (0.003)	0.000 (0.003)
Junior high school		0.006^***^ (0.002)	0.003 (0.003)	0.003 (0.003)
Senior high school		0.012^***^ (0.004)	0.010^**^ (0.004)	0.010^**^ (0.004)
College or above		0.035^***^ (0.011)	0.025^**^ (0.011)	0.026^**^ (0.011)
Spouse		−0.002 (0.004)	−0.002 (0.005)	−0.003 (0.005)
Wealth (in log)			0.003^***^ (0.001)	0.003^***^ (0.001)
Children				−0.001 (0.001)
Year fixed effect	NO	NO	YES	YES
Region fixed effect	NO	NO	YES	YES
Constant	0.007^*^ (0.000)	0.086^**^ (0.040)	−0.018 (0.016)	−0.018 (0.016)
Observations	21,587	19,677	16,188	16,032
*R*-squared	0.002	0.010	0.009	0.009

^***^, ^**^, ^*^, respectively, are significant at the levels of 1, 5, 10%, and the data in parentheses are values of *p*.

### Estimation results of IV model

The models may omit variables that affect both subjective life expectancy and commercial pension insurance participation rates, leading to endogenous bias in the estimates. When people make subjective estimates of their life expectancy, the longevity of their parents will be an important factor influencing their self-expectations. Studies in the literature have found that factors such as genetic diseases and genes can influence life expectancy across generation (Fu et al., 2020), and that the longer the parents live, the more confident individuals are in their subjective life expectancy judgments. Therefore, parental life expectancy is closely related to individual subjective life expectancy. At the same time, parental longevity does not directly influence an individual’s commercial insurance purchase decision, but only through the pathway of influencing an individual’s subjective life expectancy. Therefore, whether or not parents live long is a valid IV variable for individual subjective life expectancy. In this paper, we first investigate the variable of whether the parents are alive or not, and if the parents are alive, they are considered to be long-lived parents. Second, considering that some of the samples are close to 60 years old whose parents are actually older than 80 years old, the probability of survival is low. Therefore, in this paper, the sample whose parents’ actual survival age is over 80 years old is still defined as having long-lived parents.

Table 3 reports the regression results of the IV model, where column (1) reports the regression results of the first stage. The results show that whether the parents live longer is significantly related to the subjective expectation of the sample, which means that there is a significant correlation between the instrumental and endogenous variables. In addition, the F-statistic of the first stage reaches 85.02, indicating that it is reasonable to use whether the parents are long-lived as an instrumental variable, and there is no weakness of the instrumental variable. The regression results of the instrumental variables in column (2) of Table 3 show that the regression coefficient of subjective life expectancy is significantly positive. Thus, the regression results based on the IV model indicate that the higher the subjective life expectancy of the elderly, the higher the participation rate in commercial pension insurance. The OLS regression results are robust.

**Table 3:** Estimation results of IV model.

	(1)	(2)
	Subjective Life Expectancy	Commercial pension insurance participation
IV	0.047^***^ (0.005)	
Subjective Life Expectancy		0.077^*^ (0.043)
Control variables	YES	YES
Year fixed effect	YES	YES
Region fixed effect	YES	YES
Constant	0.156^***^ (0.037)	−0.021 (0.019)
Observations	16,398	16,391
*F* value	85.02	

Robust standard errors are reported in parentheses. ^***^, ^**^, ^*^ denote statistical significance at the 1%, 5%, and 10% level, respectively.

### Heterogeneity analysis

Analysis of Table 2 reveals that, in general, as individuals’ subjective life expectancy increases, their participation rate in commercial pension insurance increases significantly. Risk attitudes also affect the demand for commercial insurance, and they may differ across groups (Cutler et al., 2008). We next conduct further subgroup regressions to explore the heterogeneity of the effect of subjective life expectancy on commercial pension insurance participation across groups from the perspectives of respondents’ gender, education, hukou, and wealth.

In terms of gender heterogeneity, each standard deviation increase in subjective life expectancy for women is significantly associated with a 1% point increase in their commercial pension insurance participation rate. In contrast, the effect of subjective life expectancy on men’s commercial pension insurance participation is not significant (Table 4). We speculate that this may stem from gender differences in residents’ health preferences and risk attitudes. Compared to women, men are generally overconfident about their health and finances, and thus lack the incentive to save preventively. Men are less likely to plan for their retirement even when their life expectancy increases. Women, on the other hand, are more cautious and place more emphasis on short- and long-term planning for their assets.

**Table 4:** Gender heterogeneity.

	(1)	(2)	(3)	(4)
	Male		Female	
	OLS	IV	OLS	IV
Subjective Life Expectancy	0.007 (0.005)	0.002 (0.065)	0.010^**^ (0.004)	0.133^**^ (0.066)
Control variables	YES	YES	YES	YES
Year fixed effect	YES	YES	YES	YES
Region fixed effect	YES	YES	YES	YES
Observations	7,443	7,443	8,589	8,589
*R*-squared	0.008	0.008	0.012	0.012

Robust standard errors are reported in parentheses. ^***^, ^**^, ^*^ denote statistical significance at the 1%, 5%, and 10% level, respectively.

Similarly, there are urban–rural differences in the impact of subjective life expectancy on commercial pension insurance participation (Table 5). The commercial insurance market development is at an early stage in China, with business concentrated in urban areas. It exhibits a significant increase of 3.7% points in commercial pension insurance participation for a one standard deviation increase in the subjective life expectancy of urban residents. In contrast, in the sample of rural residents, subjective life expectancy has no significant effect on commercial pension insurance participation. This may be because urban residents have higher income and better asset allocation ability compared to rural residents. It also implies that rural areas will be an important market to be explored for commercial insurance companies in China, and the demand for commercial insurance among rural residents should be fully stimulated.

**Table 5:** Urban–rural heterogeneity.

	(1)	(2)	(3)	(4)
	Urban		Rural	
	OLS	IV	OLS	IV
Subjective Life Expectancy	0.005 (0.004)	0.012 (0.052)	0.014^**^ (0.007)	0.193^**^ (0.092)
Control variables	YES	YES	YES	YES
Year fixed effect	YES	YES	YES	YES
Region fixed effect	YES	YES	YES	YES
Observations	9,535	9,535	6,497	6,497
*R*-squared	0.006	0.005	0.011	0.011

Robust standard errors are reported in parentheses. ^***^, ^**^, ^*^ denote statistical significance at the 1%, 5%, and 10% level, respectively.

Education increases one’s awareness of risk and ability to deal with risk, and with higher education level comes higher ability to manage risk. We group our sample according to education level, with primary school and below as a group and senior school and above as a group. Since we select a sample of elderly people around 45–60 years old, the average years of education in China are low for all of this group during their student years, and most of them have only primary school education. The regression results support our conjecture. In the high-education group, the participation rate in commercial pension insurance significantly increases with the increase in subjective life expectancy. However, in the low-education group, where residents are less risk-aware, subjective life expectancy has no effect on the participation rate of commercial pension insurance (Table 6).

**Table 6:** Education heterogeneity.

	(1)	(2)	(3)	(4)
	Education_low		Education_high	
	OLS	IV	OLS	IV
Subjective Life Expectancy	0.007 (0.005)	−0.008 (0.071)	0.011^**^ (0.005)	0.115^*^ (0.061)
Control variables	YES	YES	YES	YES
Year fixed effect	YES	YES	YES	YES
Region fixed effect	YES	YES	YES	YES
Observations	6,778	6,778	9,254	9,254
*R*-squared	0.005	0.003	0.009	0.008

Robust standard errors are reported in parentheses. ^***^, ^**^, ^*^ denote statistical significance at the 1%, 5%, and 10% level, respectively.

Lastly, we also group the sample using household wealth indicators. The results show that the richer the household, the more significantly the commercial pension insurance participation rate is associated with subjective life expectancy, while in the low-income group, there is no significant effect of individual subjective life expectancy on commercial pension insurance participation (Table 7).

**Table 7:** Wealth heterogeneity.

	(1)	(2)	(3)	(4)
	Wealth_low		Wealth_high	
	OLS	IV	OLS	IV
Subjective Life Expectancy	0.002 (0.003)	0.022 (0.047)	0.036^***^ (0.010)	0.290^*^ (0.163)
Control variables	YES	YES	YES	YES
Year fixed effect	YES	YES	YES	YES
Region fixed effect	YES	YES	YES	YES
Observations	12,550	12,550	3,482	3,482
*R*-squared	0.006	0.004	0.014	0.012

Robust standard errors are reported in parentheses. ^***^, ^**^, ^*^ denote statistical significance at the 1%, 5%, and 10% level, respectively.

## Discussion

Based on the subjective survival probabilities answered by respondents in the data of CHARLS 2011, 2013, 2015, and 2018, Figure 2 shows the subjective survival probabilities of respondents in relation to their age, while comparing them to the objective survival probability curves. It can be found that respondents under 60 years old generally underestimate their probability of survival compared to the objective survival probability curve.^2^ Thus, in the context of a gradual increase in average life expectancy, an underestimation of subjective life expectancy may reduce the amount of assets allocated to cope with retirement life, thus increasing the likelihood of exposure to economic crisis in old age. The underestimation of subjective life expectancy may be related to the reference factors used by respondents in their projections. People tend to predict the length of their lives by the longevity of their parents or grandparents, which is associated with family genetics, while the average family life expectancy is also an important reference value for individuals’ subjective life expectancy. However, human life expectancy will continue to increase with the development of the economy, the improvement of living standards, and the advancement of medical technology, which also imposes higher requirements on financial planning for post-retirement. Therefore, improving residents’ subjective perception of life expectancy will help them planning ahead.

**Figure 2:**
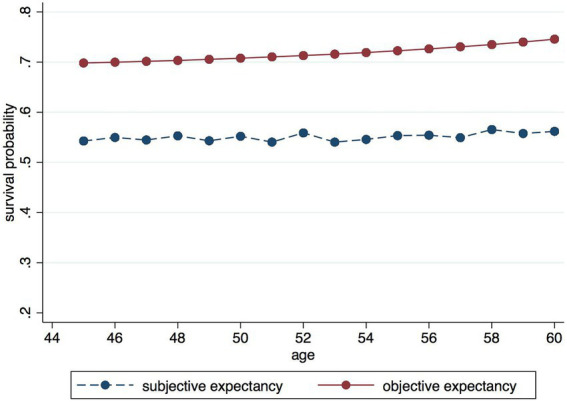
Comparison of subjective life expectancy and objective survival years.

**Figure 3:**
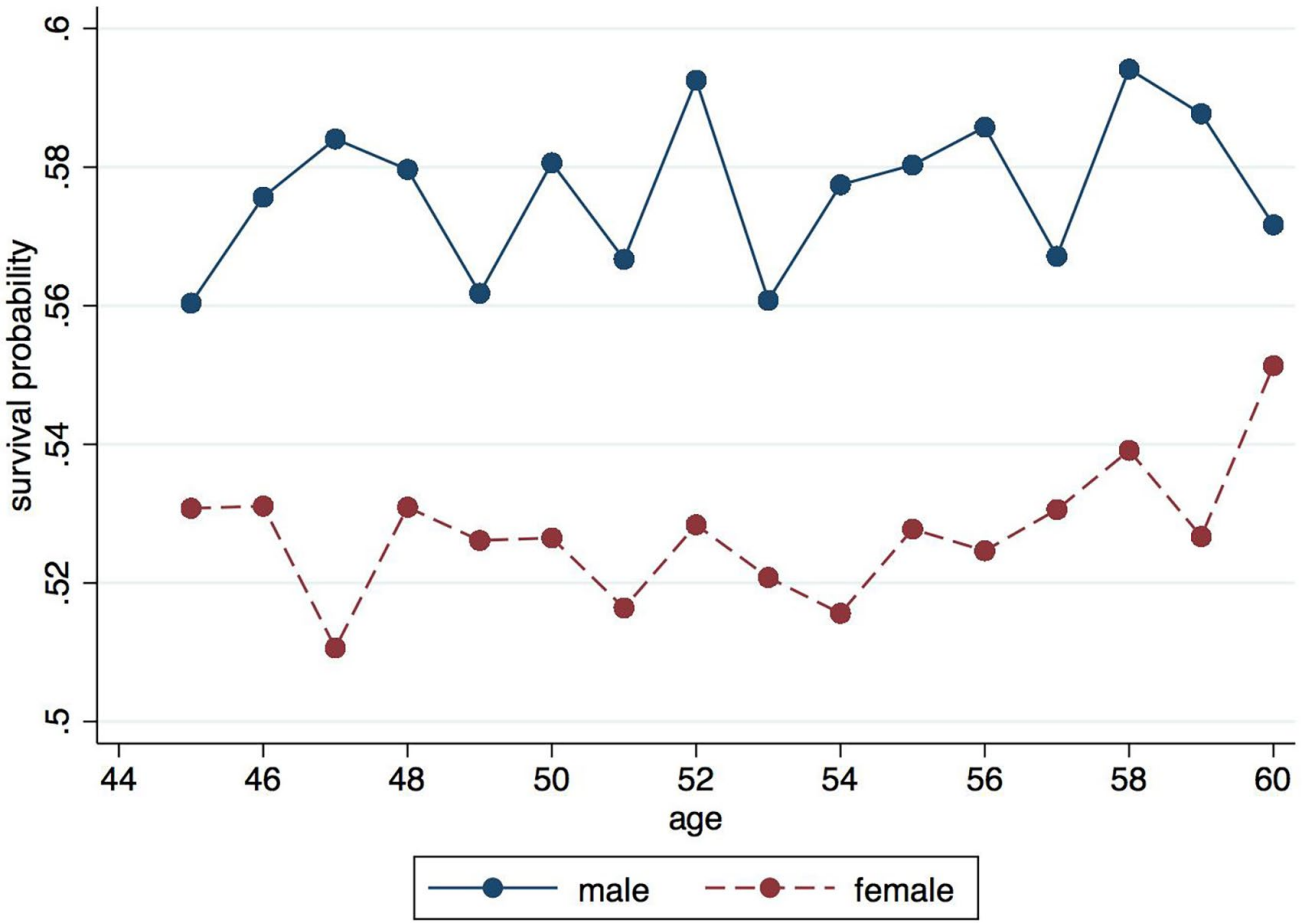
Comparison of subjective life expectancy by gender.

In addition, we also find that there are large differences in respondents’ expectations of their life expectancy by gender. As shown in Figure 3, it can be found that men show more optimism about their life expectancy, and there are more likely to overestimate their subjective life expectancy compared to women. However, this is contrary to the reality, where women tend to live longer than men on average. This is related to the natural overconfidence of men. Men are always confident about their health and future, but at the same time may not be aware enough of their own health status, which makes men show more optimism about their life expectancy and more likely to overestimate their subjective life expectancy. Our regression results, however, find that men are less adept at asset allocation and especially less skilled at planning for future assets. This reinforces our belief that men are always confident about their health and a bright future. Perhaps, men, while being optimistic, should pay more attention to the allocation of future assets in order to improve their ability to cope with economic risks in their old age.

## Conclusion

Using data from CHARLS from 2011 to 2018, this paper investigates the impact of subjective life expectancy of the elderly on commercial pension insurance participation at the micro level, providing a new perspective to explain the factors influencing commercial pension insurance participation in China. The empirical results show that each standard deviation increase in subjective life expectancy of the elderly significantly increases their commercial pension insurance participation rate by about 0.9 percentage points, and IV model estimation shows that the OLS regression results are robust. There is a significant gender difference in the effect of subjective life expectancy of the elderly on their commercial pension insurance participation, with subjective life expectancy mainly affecting women’s commercial pension insurance participation, while it has no significant effect on men’s. In addition, there is also a significant urban–rural difference in the effect of subjective life expectancy of the elderly on their commercial pension insurance participation, with an increase in subjective life expectancy significantly increasing the commercial pension insurance participation rate of urban residents, while having no significant effect on rural areas. In terms of educational heterogeneity, only respondents in the high-education group significantly increased their asset allocation in retirement when their subjective expectations increased, while respondents in the low-education group had lower commercial pension insurance participation due to their lower risk awareness. Finally, among the different wealth groups, the high wealth group significantly increased their commercial pension insurance participation when their subjective life expectancy increased, while the low wealth group had no change in commercial pension insurance participation.

Our analysis of the data collected in the CHARLS questionnaire on “subjective survival probability of respondents” reveals that there is a general underestimation of subjective survival expectations among 45–60 year olds, which may be related to their reference sample, and the specific reasons behind this are worth further investigation. In addition, men are more confident in their health and life expectancy, but do not plan carefully for retirement, the reasons, and implications of which also deserve further study.

## Data availability statement

The raw data supporting the conclusions of this article will be made available by the authors, without undue reservation.

## Author contributions

MZ and SZ carried out the study, analyzed the data, and drafted the manuscript. YW and YL were responsible for writing the literary and revising the language. RS provided the guidance for revising the manuscript. All authors contributed to the article and approved the submitted version.

## Funding

This paper was funded by the National Natural Science Foundation of China, “Health Care for the Elderly, Medical Expenditure and Savings” (71773080).

## Conflict of interest

The authors declare that the research was conducted in the absence of any commercial or financial relationships that could be construed as a potential conflict of interest.

## Publisher’s note

All claims expressed in this article are solely those of the authors and do not necessarily represent those of their affiliated organizations, or those of the publisher, the editors and the reviewers. Any product that may be evaluated in this article, or claim that may be made by its manufacturer, is not guaranteed or endorsed by the publisher.

## Footnotes

^1^The question used in the CHARLS questionnaire to ask respondents about their subjective life expectancy is: “Assuming there are five levels, with the lowest level representing the least likely and the highest level representing the most likely, how likely do you envision you will live to a certain age? 1. Almost impossible. 2. Not very likely. 3. Maybe. 4. Very likely. 5. Almost certain” (No. DA081).

^2^The objective survival probability curve was drawn from the 2010 National population Complete Life Table.
